# Impact of a low-technology simulation-based obstetric and newborn care training scheme on non-emergency delivery practices in Guatemala

**DOI:** 10.1016/j.ijgo.2015.08.009

**Published:** 2016-03

**Authors:** Anna Walton, Edgar Kestler, Julia C. Dettinger, Sarah Zelek, Francesca Holme, Dilys Walker

**Affiliations:** a School of Medicine, University of Washington, Seattle, WA, USA; b Epidemiological Research Center in Sexual and Reproductive Health, Guatemala City, Guatemala; c Department of Global Health, University of Washington, Seattle, WA, USA; d Department of Obstetrics and Gynecology and Global Health Sciences, University of California, San Francisco, San Francisco, CA, USA

**Keywords:** Emergency obstetric and newborn care, Guatemala, Maternal mortality, Neonatal mortality, Skilled birth attendants, Training

## Abstract

**Objective:**

To assess the effect of a low-technology simulation-based training scheme for obstetric and perinatal emergency management (PRONTO; Programa de Rescate Obstétrico y Neonatal: Tratamiento Óptimo y Oportuno) on non-emergency delivery practices at primary level clinics in Guatemala.

**Methods:**

A paired cross-sectional birth observation study was conducted with a convenience sample of 18 clinics (nine pairs of intervention and control clinics) from June 28 to August 7, 2013. Outcomes included implementation of practices known to decrease maternal and/or neonatal mortality and improve patient care.

**Results:**

Overall, 25 and 17 births occurred in intervention and control clinics, respectively. Active management of the third stage of labor was appropriately performed by 20 (83%) of 24 intervention teams versus 7 (50%) of 14 control teams (*P* = 0.015). Intervention teams implemented more practices to decrease neonatal mortality than did control teams (*P* < 0.001). Intervention teams ensured patient privacy in 23 (92%) of 25 births versus 11 (65%) of 17 births for control teams (*P* = 0.014). All 15 applicable intervention teams kept patients informed versus 6 (55%) of 11 control teams (*P* = 0.001). Differences were also noted in teamwork; in particular, skill-based tools were used more often at intervention sites than control sites (*P* = 0.012).

**Conclusion:**

Use of PRONTO enhanced non-emergency delivery care by increasing evidence-based practice, patient-centered care, and teamwork.

## 1 Introduction

The maternal mortality ratio (MMR) in Guatemala is one of the highest in Latin America at 120 maternal deaths per 100 000 live births [1]. Furthermore, indigenous women living in rural Guatemalan communities face the possibility of a substantially elevated MMR [2]. This situation is highlighted in the so-called “corridor of death,” a geographic area comprising four departments with large rural and indigenous populations and some of the highest MMRs in the country: Huehuetenango (MMR 226 per 100 000 live births), Alta Verapaz (MMR 207 per 100 000 live births), Quiché (MMR 196 per 100 000 live births), and San Marcos (MMR 106 per 100 000 live births) [2]. In 2010, the Guatemalan government passed the Law for Healthy Motherhood (decree 32-2010) to ensure access to safe labor and delivery care. Nonetheless, despite interventions that aim to increase institutional births, meeting Millennium Development Goal 5 (to decrease maternal mortality) remains a distant hope for these four departments owing to multilevel barriers, including poor-quality obstetric care [3], [4], [5].

The PRONTO (Programa de Rescate Obstétrico y Neonatal: Tratamiento Óptimo y Oportuno) scheme is a highly realistic, low-technology, in situ, simulation-based obstetric and perinatal emergency training program for multidisciplinary teams in low-resource settings, which has been successfully piloted and implemented in Mexico [6]. This program aims to decrease maternal and perinatal mortality through training to improve responses to the most frequent obstetric and neonatal emergencies and in the use of evidence-based practices for uncomplicated birth. The training curriculum is based on WHO standards in accordance with the Guatemalan Ministry of Health action plan [7], [8], [9], [10]. PRONTO training comprises two modules [6]. Module I (16 hours; six simulations) covers teamwork, evidence-based practices for uncomplicated birth, obstetric hemorrhage, and neonatal resuscitation. Module II (8 hours; three simulations) occurs 2–3 months later and covers pre-eclampsia, eclampsia, chorioamnionitis, and shoulder dystocia. Institutional sustainability and understanding traditional birth practices were added to the PRONTO curriculum specifically for use in Guatemala [11].

The aim of the present study was to assess the effect of PRONTO training on three domains of clinical action during uncomplicated delivery: use of evidence-based practices, provision of culturally sensitive and patient-centered care, and use of communication and teamwork skills.

## 2 Materials and methods

The present cross-sectional birth observation study was conducted to assess the effect of the PRONTO component of a package of community- and facility-level interventions on provider practices during non-emergency delivery. The package included PRONTO training, a social marketing campaign, and professional midwives serving as liaisons between clinics and their communities. The impact of the package as a whole was assessed as part of a pair-matched, cluster-randomized trial, which was implemented in 2012 in 30 primary-care clinics in Alta Verapaz, Huehuetenango, Quiché, and San Marcos. Full details of the protocol have been published previously [12]. PRONTO training began on July 30, 2012, with collection of follow-up data completed by September 1, 2013.

The present cross-sectional study focused on the effect of the PRONTO component of the intervention on uptake of specific practices during uncomplicated deliveries. Consequently, a 6-week study was conducted with a convenience sample of the intervention and control clinics between June 28 and August 7, 2013. Approval was obtained from both the Guatemalan Ministry of Health, Guatemala City, and the institutional review board of the University of Washington, Seattle (41922-E/K). The original randomized study, which included PRONTO training, was registered at clinicaltrials.gov (NCT01653626) [12].

Owing to data collection time constraints, only 18 of the original 30 clinics were selected for inclusion in the present study. The goal was to observe three to five births within a period of 1–5 days in each clinic. The final nine clinic pairs were selected to balance the need for high delivery volume and low expected travel time between facilities, ensuring equal representation from each of the four departments (two clinic pairs per department). Additionally, a third clinic pair was selected from Alta Verapaz because a large proportion of the sites recruited in the original study was from this department to reflect both its large population and large geographic area.

On arrival at each clinic, fieldworkers (including A.W.) met with the director to describe the project and obtain consent. An information sheet written in Spanish about the proposed research activities was provided to both staff and patients. Only pregnant women who spoke Spanish or a native language shared by staff attending the birth were included in the present study. No personal identifiers were collected to maintain confidentiality of the women, newborns, and providers.

Study data were collected by two fieldworkers (one of whom was A.W.). The fieldworkers had trained together for the purposes of the present study at a large public hospital in Guatemala City, using a standardized observation form to collect data during births. During the training period, the fieldworkers independently completed a form for each delivery observed until their responses were consistent with an expert observer (assuring correct classification of behaviors and practices) and with each other (assuring interobserver reliability). The birth observation form has been used in other countries where PRONTO has been implemented. The form, which is the same used to collect study data, included 63 data points covering demographics, evidence-based practices, cultural sensitivity and patient-centered care, and teamwork and communication.

Demographic information included age, length of pregnancy, 1-minute and 5-minute Apgar scores, parity, type of provider attending the delivery, presence of a birth companion, time of delivery, and observation time. Clinic characteristics included: birth volume (deliveries per month); perinatal mortality (deaths per month); number of healthcare personnel by type; availability of oxytocin, magnesium sulfate, and antibiotics; and availability of Doppler and central sterilization. These baseline clinic data were collected over 4 months before initiating module I of the PRONTO training as part of the large randomized study and are included here to provide a more complete picture of the study setting. All study variables are defined in Table 1 [7], [8], [9], [11], [13], [14].

The data were analyzed using STATA version 12.1 (StataCorp, College Station, TX, USA). One-tailed and two-tailed *t* tests with 40 degrees of freedom were used to assess between-group differences in the proportion of each practice used. *P* < 0.05 was considered statistically significant.

## 3 Results

A total of 42 births occurred during the present study period, 25 in eight of the intervention clinics and 17 in eight of the control clinics. Owing to time limitations and low delivery volumes, no births were observed in one intervention clinic and in one control clinic. In all, 20 different primary providers were observed in the intervention clinic group and 15 in the control clinic group.

The clinic and delivery characteristics are presented in Table 2. No statistically significant differences were observed in the baseline clinic characteristics between the intervention and control groups. Furthermore, no statistically significant between-group differences were observed in the delivery characteristics, with the exception of length of pregnancy. The mean length of pregnancy was 39 ± 0.8 weeks in the intervention clinic group and 38 ± 1.8 weeks in the control clinic group (*P* = 0.021). However, in both groups, the mean length of pregnancy was considered to be “term” (defined as at least 37 weeks). All recorded deliveries were non-emergent, or uncomplicated, live births, with the exception of one unexpected macerated stillbirth at a control site. No attempt was made to resuscitate. Medical doctors (predominantly general practitioners) attended most births. One intervention clinic employed obstetrician–gynecologists, who attended three of the births in this group. Professional or auxiliary nurses attended births when no physicians were present. Deliveries in both groups occurred in the presence of companions, such as traditional birth attendants and family members; however, no significant between-group difference was noted for this variable.

Clinic use of evidence-based delivery and newborn practices is shown in Table 3. Both groups exhibited high usage rates (> 50%) of these practices, with the exception of skin-to-skin contact, which was 42% in the intervention group (n = 10) and 31% in the control group (n = 5). Although not a recommended practice when access to cesarean delivery is unavailable, oxytocin to augment second stage of delivery was also performed in less than 50% of deliveries in both groups. Intervention clinics implemented a mean of 4.8 ± 1.0 of the seven evidence-based practices (active management of third stage of labor serving as one composite variable) versus 3.4 ± 1.2 in the control clinics (*P* < 0.001). All evidence-based practices were observed more frequently in the intervention clinics than the control clinics, including immediate mother-to-neonate contact (*P* = 0.023) and covering the neonate within 30 seconds of delivery (*P* = 0.003). Although intervention clinics tended to introduce the neonate to the breast within 1 hour of delivery, examine the placenta, and delay cord clamping more often than did control clinics, the between-group differences were not statistically significant. Also, whereas providers in intervention clinics completed all three steps of active management of the third stage of labor more often than their counterparts in control clinics (*P* = 0.015), no difference was observed in appropriate oxytocin use (*P* = 0.792) or the timing of oxytocin administration (*P* = 0.301).

A statistically significant between-group difference was detected in the provision of patient-centered and culturally sensitive care (Table 4). Intervention sites implemented a mean of 2.7 ± 1.4 of six behaviors compared with 1.9 ± 1.3 for control sites (*P* = 0.040). Staff addressing patients by name was more frequent in intervention clinics than in control clinics (13 [52%] of 25 vs 4 [24%] of 17; *P* = 0.034), as was the provision of privacy (23 [92%] vs 11 [65%]; *P* = 0.014). Information was provided to patients by staff on patient requests for all 15 relevant births in intervention clinics, but only 6 (55%) of 11 births in control clinics (*P* = 0.001). Differences in freedom of delivery position and movement were not statistically significant, although this practice was universally low (< 25%). Similarly, responding to requests (e.g. to make traditional tea for a patient) made by patient’s companions did not differ; however, the frequency of patients or companions making such requests was low. The staff in the intervention clinics employed positive verbal and nonverbal communication in all 25 observed births, compared with 13 (76%) births in control clinics (*P* = 0.005). Negative communication was directed toward the patient during delivery in both groups (≥ 65%), with no significant difference detected (*P* = 0.687).

Intervention clinics were more likely than control clinics to use teamwork and communication tools (Table 5). Clinicians in intervention clinics implemented a mean of 9.4 ± 1.5 of these 12 techniques compared with 8.0 ± 2.4 in control clinics (*P* = 0.012). Specific communication techniques were used more frequently in intervention clinics than in control clinics, including situation–background–assessment–recommendation (5 [83%] of 6 births vs 4 [36%] of 11; *P* = 0.035), check backs (12 [48%] of 25 births vs 2 [13%] of 16; *P* = 0.009), and thinking out loud (21 [84%] of 25 vs 9 [53%] of 17; *P* = 0.014). Teams in intervention facilities also implemented situation monitoring more often than those in control facilities, including reporting the status of the patient during labor to team members (23 [92%] vs 13 [76%]; *P* = 0.083) and communicating with each other about their work (19 [76%] vs 10 [59%]; *P* = 0.124). All clinics exhibited almost universal mutual support and leadership techniques; however, intervention clinic staff were more likely than control clinic staff to ask for help (25 [100%] vs 15 [88%]; *P* = 0.041) and leaders in intervention facilities were more likely than their counterparts in control facilities to allow staff members to speak up (23 [96%] vs 14 [82%]; *P* = 0.080).

## 4 Discussion

The findings of the present study suggested that PRONTO training positively influenced practices during non-emergency labor, delivery, and immediate postpartum care. Overall, skilled birth attendants in the intervention sites implemented more evidence-based practices, provided more patient-centered care, and used more teamwork and communication tools than did those in the control sites. Other simulation-based programs have been found to improve management of complications [15], [16], [17], [18] and provider performance and teamwork during delivery and postpartum care [19], [20]. Nevertheless, PRONTO is unique in that is offers a highly realistic, low-cost, in situ simulation, technique-based focus on both the mother and her newborn. In addition, PRONTO incorporates evidence-based teamwork and communication tools, with an emphasis on cultural humility and kind and respectful care.

Evidence-based practices were highly used in both the intervention and control clinics, with the exception of skin-to-skin contact. As part of the original PRONTO study [12], providers completed a detailed surveillance form during and after each delivery that had been adapted from the WHO near-miss tool and validated for use in Guatemala [21]. This form included provider self-reported use of evidence-based practices, delivery complications, and critical interventions. The high rate of evidence-based practice recorded in the present cross-sectional study was consistent with preliminary analysis of the original study results (unpublished data). These results were promising and suggested the potentially positive effect of incorporating the surveillance form into PRONTO as it seemed to provide a trigger for using evidence-based practices, essentially guiding providers through the correct care actions as a type of self-audit [21].

Although PRONTO training had a positive effect on the care provided during non-emergency labor and delivery in Guatemalan primary level facilities, the present study found that the birth attendants did not universally implement certain fundamental components of non-emergency delivery care. Problem areas included a high proportion of negative communication during deliveries, inappropriate use of oxytocin to augment the second stage of labor given the inability to provide cesarean delivery in the case of failed augmentation, and underutilization of skin-to-skin contact and freedom of movement and delivery position. These results indicated that a need exists to incorporate teachings on non-emergency delivery care into national efforts to train birth attendants working in low-resource settings in emergency management.

The findings of the present study were generally encouraging; however, there are limitations inherent to the cross-sectional, non-blinded, convenience sampling nature of the design. Preintervention birth observations were not performed; therefore, the observed differences could not be attributed solely to PRONTO training. Nonetheless, discussions with several participants suggested that they attributed changes in personal behavior to the training that they had received. Additionally, findings from the process evaluation of PRONTO were indicative of substantial improvement in trained provider knowledge and self-efficacy in the intervention clinics, which might be linked to changes in practice.

The 18 clinics included in the present study represented a convenience sample designed to maximize the number of deliveries observed in a short period of time. This approach was unlikely to bias the findings because the clinics displayed similar baseline characteristics and they were all sampled from the original randomized matched-pair cluster study [12]. However, the data obtained might be generalized to only high-volume primary level facilities in Guatemala. The short timeframe allowed for birth observations (6 weeks) resulted in a small number of deliveries (n = 42). Nonetheless, given a sample size of 25 observations in the intervention facilities and 17 in the control facilities, the present study was powered (α 0.05; power 85%) to detect a greater than 25% difference in the use of active management of the third stage of labor, assuming a standard deviation of 0.3.

Blinding was impossible in the present study owing to the design of the original study, which included an extensive marketing campaign with banners and other materials that were easily seen upon arrival at intervention sites [12]. The inability to formally blind fieldworkers might have led to bias in data collection, especially for those variables that require subjective assessment, including the areas of leadership and mutual support. However, these actions were identified in most deliveries observed, which suggested that minimal bias had occurred. Finally, participants knew that they were being observed and the presence of the fieldworker could have unintentionally influenced their behaviors. The practices exhibited during the birth observations could have differed from standard of care. In addition, providers who received the PRONTO training might have been motivated to practice the techniques that they learned during the sessions with an outside observer.

Despite these limitations, the strengths of the original study [12] probably counteract many of the potential biases of the present study. In addition, the method of birth observation enabled a more direct and standardized approach to the collection of behavioral data than self-report.

In conclusion, the use of PRONTO could benefit non-emergency delivery care in Guatemala. Further investigation is, therefore, warranted to rigorously assess the impact of such simulation training on emergency and non-emergency birth practices in this country.

## Figures and Tables

**Table 1:** Variables included in the birth observation form.

Variable	Definition
Evidence-based practices [7], [8], [9]	
Skin-to-skin contact between mother and newborn	Newborn is placed on the mother’s bare skin immediately following delivery and before cutting of the umbilical cord. Drying and covering of the newborn might occur while it is laid on the mother.
Drying and covering the newborn	Wiping the newborn with a towel and swaddling within 30 s of delivery.
Examination of the placenta	Primary provider examines the placenta for completeness and to ensure that no remnants were left in the uterus.
Introduction to the breast within 1 h of birth	The mother and newborn are left together and encouraged to initiate breastfeeding.
Delayed cord clamping	Waiting at least 1 min after delivery before clamping and cutting the umbilical cord.
Active management of the third stage of labor	Composite variable. Providers must complete all three of the following items: (1) Intramuscular injection of 10 IU of oxytocin administered to the mother within 1 min of giving birth; (2) Controlled traction on the umbilical cord for delivery of the placenta with suprapubic countertraction; (3) Uterine massage after delivery of the placenta.
Time to oxytocin injection	Time (min) elapsed between delivery to intramuscular injection of oxytocin.
Culturally sensitive and patient-centered care [11]	
Provider refers to the patient by her name	Provider asks the patient what she would like to be called; uses the patient’s name at least half of the time instead of using generic terms such as “Miss.”
Provider gives the patient all information requested	If the patient asks questions, the team members respond with an appropriate answer. Mark the form as “NA” if the patient did not ask anything.
Provider allows the patient freedom of movement or delivery position	Providers either ask the patient whether she has a preference for the position in which she would like to deliver or they allow the patient to move. Mark the form as “No” if providers blatantly restricted patient movement.
Provider ensures patient privacy	Providers ensure curtains or doors are closed and/or that the patient is covered. Mark the form as “No” if the patient was left exposed in the triage and/or waiting rooms.
Team acknowledges at least one request from the patient and/or her companion(s)	Providers allow patients and their families to bring in blankets, prepare tea etc. if requested. Mark the form as “NA” if the patients and/or companion(s) did not request anything.
Positive verbal and non-verbal communication	Providers use encouraging words or phrases (e.g. “You can do it,” “You are almost there,” or “You are doing great”); kind tone; eye contact; and/or supportive touch.
Negative verbal and non-verbal communication	Providers use demeaning or disrespectful words or phrases (e.g. “Stop crying” or “You are taking forever”); condescending tone; are dismissive; ignore the patient; make judgmental statements about the patient within hearing distance; do not make eye contact with the patient; and/or throw items at the patient.
Teamwork, leadership, and communication [13], [14]	
Situation–background–assessment–recommendation	Structured communication tool for hand-off between providers.
Check backs	Closed-loop communication between at least two providers. One provider requests or states something, the receiving provider repeats it, and the original provider confirms or corrects it.
Thinking out loud	All team members vocalize thought process behind actions immediately before or during actions.
Team communicates with the patient	Providers keep the patient updated and informed of what they are doing and why they are doing it.
Team members report the patient’s health status to each other	Providers constantly update the rest of the team about any new findings or updates, such as blood pressure, contractions, cervical dilation, or fetal heart rate.
Team members interact with each other about their work	Providers communicate openly about their actions and give constructive feedback to each other when appropriate.
Team members ask for help	Providers openly and proactively request assistance from others when needed.
Team members assist each other	Team members proactively identify team needs and act accordingly to meet those needs.
Team members identify errors	If errors occur, providers acknowledge them immediately and openly and do not deny or blame others. Mark the form as “NA” if no errors were observed.
Leader guides the team’s work	If a team leader is identified, he or she clearly guides the activities and sets priorities of the team. Mark the form as “NA” if no clear leader identified.
Leader delegates tasks	If a team leader is identified, he or she confidently and appropriately delegates tasks to capable team members. Mark the form as “NA” if no clear leader identified.
Leader fosters an environment in which members express themselves	Team members freely express concerns, questions, ideas, and suggestions without fear of reprisal or judgment. Mark the form as “NA” if no clear leader identified.

Abbreviation: NA, not applicable.

**Table 2:** Clinic and delivery characteristics.^a^

Variable	Intervention clinics	Control clinics	*P* value^b^
Clinic characteristics	n = 8	n = 8	
Birth volume, deliveries/mo	430 ± 96	338 ± 42	0.389
Perinatal mortality, deaths/mo	4.5 ± 0.9	6.3 ± 1.4	0.300
Personnel			
Medical doctor	4.0 ± 0.7	3.4 ± 0.4	0.452
Professional nurse	3.4 ± 0.4	4.4 ± 0.6	0.179
Auxiliary nurse	13.3 ± 1.5	14.1 ± 1.1	0.417
Medication available			
Antibiotics	8 (100)	8 (100)	> 0.99
Magnesium sulfate	4 (50)	3 (38)	0.614
Oxytocin	8 (100)	8 (100)	> 0.99
Equipment available			
Doppler	7 (88)	6 (75)	0.522
Central sterilization	7 (88)	6 (75)	0.522
Delivery characteristics	n = 25	n = 17	
Patient age, y	25.6 ± 9.0	25.2 ± 8.4	0.883
Parity	2.4 ± 3.0	1.8 ± 2.4	0.517
Gestational age, wk	39.2 ± 0.8	38.2 ± 1.8	0.021
Apgar score			
1 min	8.3 ± 0.75	7.5 ± 2.2	0.101
5 min	9.0 ± 0.45	8.4 ± 2.2	0.233
Observation time, min	47 ± 34	50 ± 32	0.768
Births during the day shift	15 (60)	13 (76)	0.278
Birthing companion present	10 (40)	5 (30)	0.247
Attending provider			
Medical doctor	15 (60)	10 (59)	0.941
Professional nurse	5 (20)	2 (12)	0.494
Auxiliary nurse	5 (20)	5 (29)	0.494

a Values given as mean ± standard deviation or number (percentage), unless indicated otherwise.

b Student *t* test.

**Table 3:** Use of evidence-based delivery and newborn practices at the intervention and control clinics.^a^

Practice	Births in intervention clinics (n = 25)	Births in control clinics (n = 17)	*P* value^b^
Immediate contact between mother and neonate^c^	24/25 (96)	12/16 (75)	0.023
Skin-to-skin contact between mother and neonate^c^	10/24 (42)	5/16 (31)	0.259
Neonate covered and dried within 30 s^c^	22/25 (88)	8/16 (50)	0.003
Introduction to breast within 1 h^c^	24/25 (96)	13/16 (81)	0.063
Placenta examined	23/25 (92)	13/17 (76)	0.083
Delayed cord clamping > 1 min^c^	22/25 (88)	12/16 (75)	0.083
Active management of the third stage of labor^c^	20/24 (83)	7/14 (50)	0.015
Oxytocin administered during the third stage of labor	24/25 (96)	17/17 (100)	0.792
Oxytocin administered to augment the second stage of labor	4/25 (16)	3/17 (18)	0.446
Mean time to oxytocin injection, min	1.1 ± 1.1	1.3 ± 0.9	0.301
No. of evidence-based practices implemented	4.8 ± 1.0	3.4 ± 1.2	< 0.001

a Values given as number/total number (percentage) or mean ± standard deviation, unless indicated otherwise.

b Student *t* test.

c Births without the opportunity for a specific action were excluded from that activities analysis (e.g. in the case of a macerated stillbirth in one of the control sites).

**Table 4:** Use of patient-centered care at the intervention and control clinics.^a^

Practice	Intervention clinics (n = 25)	Control clinics (n = 17)	*P* value^b^
Patient referred to by name	13 (52)	4 (24)	0.034
All information requested was provided to the patient^c^	15/15 (100)	6/11 (55)	0.001
Movement and freedom of delivery position allowed	6 (24)	2 (12)	0.167
Privacy given to the patient	23 (92)	11 (65)	0.014
Acknowledgement of a request from the patient and/or her birthing companion^c^	9/12 (75)	8/14 (57)	0.180
Positive communication	25 (100)	13 (76)	0.005
Negative communication	18 (72)	11 (65)	0.687
No. of patient-centered care practices implemented	2.7 ± 1.4	1.9 ± 1.3	0.040

a Values given as number/total number (percentage), number (percentage), or mean ± standard deviation, unless indicated otherwise.

b Student *t* test.

c Births without the opportunity for a specific action were excluded from that activities analysis (e.g. when companions did not make any requests).

**Table 5:** Use of teamwork and communication practices at the intervention and control clinics.^a^

Practice	Intervention clinics (n = 25)	Control clinics (n = 17)	*P* value^b^
Effective communication			
Situation–background–assessment–recommendation^c^	5/6 (83)	4/11 (36)	0.035
Check backs	12 (48)	2/16 (13)	0.009
Thinking out loud	21 (84)	9 (53)	0.014
Situation monitoring			
The team communicates with the patient	25 (100)	15 (88)	0.041
Reports patient’s health status to team members	23 (92)	13 (76)	0.083
Interact with each other about their work	19 (76)	10 (59)	0.124
Mutual support			
Team members assist each other	25 (100)	17 (100)	> 0.99
Team members ask for help	25 (100)	15 (88)	0.041
Team members identify errors if they occur^c^	10/10 (100)	5/6 (83)	0.104
Leadership			
Leader guides team activities^c^	23/24(96)	16 (94)	0.404
Leader delegates^c^	24/24 (100)	16 (94)	0.120
Leader allows members to speak up^c^	23/24 (96)	14 (82)	0.080
Total number of teamwork and communication practices implemented	9.4 ± 1.5	8.0 ± 2.4	0.012

a Values given as number/total number (percentage), number (percentage), or mean ± standard deviation, unless indicated otherwise.

b Student *t* test.

c Births without the opportunity for a specific action were excluded from that activities analysis (e.g. if no errors occurred).
